# Clinical characteristics and outcomes of pregnant women with COVID-19 and the risk of vertical transmission: a systematic review

**DOI:** 10.1007/s00404-020-05889-5

**Published:** 2020-12-01

**Authors:** Jianhua Chi, Wenjian Gong, Qinglei Gao

**Affiliations:** 1 Cancer Biology Research Center (Key Laboratory of the Ministry of Education), Tongji Hospital, Tongji Medical College, Huazhong University of Science and Technology, 1095 Jiefang Ave, Wuhan, Hubei China; 2 Department of Gynecology and Obstetrics, Tongji Hospital, Tongji Medical College, Huazhong University of Science and Technology, Wuhan, Hubei China

**Keywords:** COVID-19, Delivery, Infants, Pregnancy, SARS-CoV-2, Vertical transmission

## Abstract

**Purpose:**

This systematic review summarizes the clinical features and maternal–infant outcomes of 230 pregnant women (154 patients gave birth) infected with COVID-19 and their 156 infants, including the possibility and evidence of vertical transmission.

**Methods:**

An electronic search of PubMed, Embase, Medline, MedRxiv, CNKI, and the Chinese Medical Journal Full Text Database following PRISMA guidelines was performed through April 18, 2020. Search terms included COVID-19, SARS-CoV-2, pregnant women, infants, and vertical transmission.

**Results:**

A total of 230 women with COVID-19 (154 deliveries, 66 ongoing pregnancies, and 10 abortions) and 156 newborns from 20 eligible studies were included in this systematic review. A total of 34.62% of the pregnant patients had obstetric complications, and 59.05% of patients displayed fever. Lymphopenia was observed in 40.71% of patients. A total of 5.19% of women received mechanical ventilation. Seven women were critically ill. One mother and two newborns died. A total of 24.74% of newborns were premature. Five newborns’ throat swab tests of SARS-CoV-2 were positive, all of which were delivered by cesarean section. For eight newborns with negative throat swab tests, three had both elevated IgM and IgG against SARS-CoV-2. Nucleic acid tests of vaginal secretions, breast milk, amniotic fluid, placental blood, and placental tissues were negative.

**Conclusion:**

Most pregnant patients were mildly ill. The mortality of pregnant women with COVID-19 was lower than that of overall COVID-19 patients. Cesarean section was more common than vaginal delivery for pregnant women with COVID-19. Premature delivery was the main adverse event for newborns. The vertical transmission rate calculated by SARS-CoV-2 nucleic acid tests was 3.91%. Serum antibodies against SARS-CoV-2 should be tested more frequently, and multiple samples should be included in pathogenic testing.

## Introduction

Coronavirus disease 2019 (COVID-19), caused by severe acute respiratory syndrome coronavirus 2 (SARS-CoV-2), was characterized as a pandemic by the World Health Organization on March 11, 2020 [1]. It is believed that pregnant women are more susceptible to virus infections due to immunologic and anatomic alterations [2]. Pregnant women with severe acute respiratory syndrome (SARS) or middle east respiratory syndrome (MERS) have serious adverse outcomes, such as maternal deaths and premature births, while no evidence of vertical transmission has been found [3]. As more articles have described the clinical characteristics and outcomes of pregnant women with COVID-19, our understanding of how COVID-19 may influence pregnant women continues to improve. This systematic review aims to summarize the clinical characteristics and maternal–infant outcomes of pregnant women with COVID-19, especially the possibility of vertical transmission.

## Methods

### Search strategy and selection criteria

An electronic search of PubMed, Embase, Medline, MedRxiv, CNKI (China National Knowledge Infrastructure), and the Chinese Medical Journal Full Text Database (http://journal.yiigle.com/) was performed following Preferred Reporting Items for Systematic reviews and Meta-Analyses (PRISMA) guidelines [4] through to April 18, 2020. Search terms were (COVID-19 OR 2019-nCoV OR SARS-CoV-2 OR 2019 Novel coronavirus) AND (pregnant women OR Maternal OR parturient OR mothers OR Infant OR Newborn OR neonate OR Infectious Disease Transmission, Vertical).

The titles and abstracts in the search results were independently reviewed by two authors (Jianhua Chi and Wenjian Gong) to determine whether the article was relevant to our research topic. The full texts of the literature we identified were further searched, and we read the articles to identify those eligible that ultimately met the inclusion and exclusion criteria. The inclusion criteria were as follows: 1, pregnant women were diagnosed with COVID-19 by dual fluorescence polymerase chain reaction (PCR) or quantitative real-time polymerase chain reaction (qRT-PCR), or were clinically diagnosed cases according to the latest clinical guidelines at the time of the article’s publication; 2, articles that described clinical characteristics and maternal and newborn outcomes. The exclusion criteria included, 1, duplicated cases and 2, newborns who tested positive for COVID-19 but lived with COVID-19 relatives to avoid confounding factors when assessing vertical transmission. We abandoned duplicated cases by referring to the birthplace, times of admission and delivery, specific maternal characteristics of cases, as well as dates and authors of publications to eliminate potential overlap of cases.

### Data extraction, assessment of risk of bias, and data synthesis

The following variables were extracted from eligible literature: study type, time, region, inclusion criteria, sample size, epidemiological history of mothers and newborns related to COVID-19, delivery mode (cesarean section/vaginal delivery), cesarean section indications, fetal sex (male/female), gestational age, birth weight, clinical diagnosis, imaging, oxygen therapy, antiviral treatment, breastfeeding, maternal and newborn outcomes, case admission time and hospital name, number of pregnant women, clinical characteristics and outcomes of pregnant women, number of newborns, characteristics and outcomes, method of diagnosis and number of positive diagnoses. To avoid bias, the authors received training, established a consensus, read all articles separately, and discussed any ambiguities.

We adopted an approach specially used for case reports and case series that was proposed by Murad et al. [5] to evaluate the quality of the included articles. According to this method, two authors read all articles and considered four aspects, selection, ascertainment, causality, and reporting.

## Results

### Study selection and characteristics

**Fig. 1:**
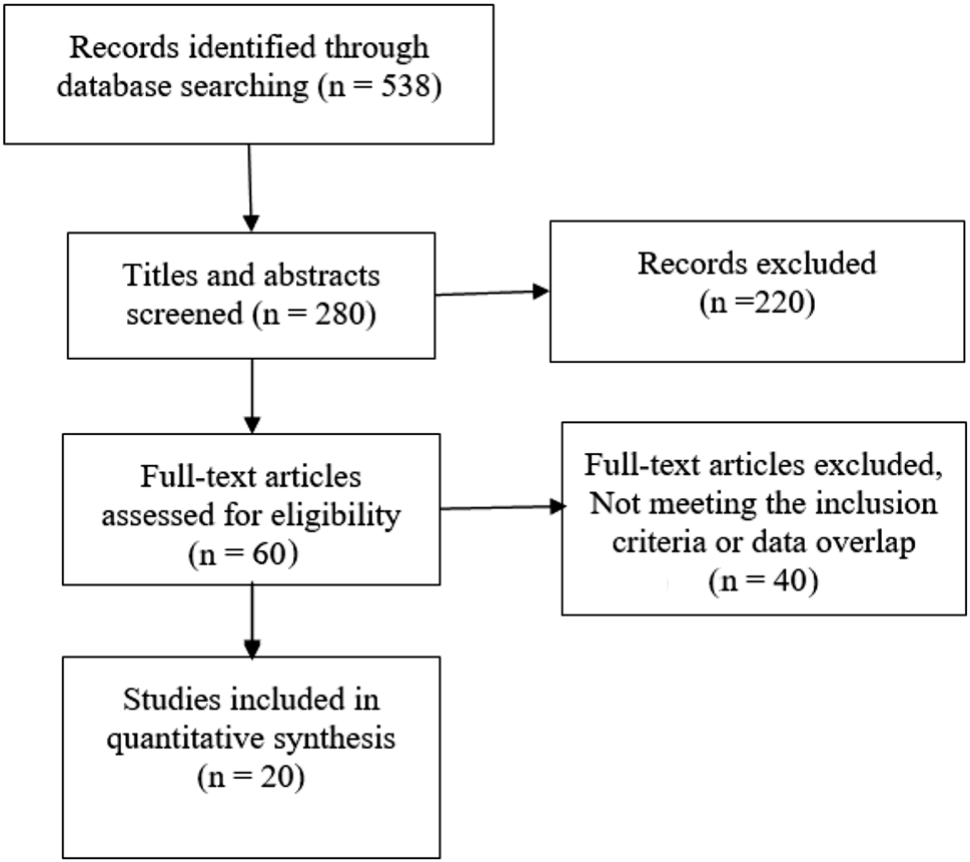
PRISMA flowchart of included studies

A preliminary search returned 538 articles. After reading the titles and abstracts, 60 articles were chosen, and the others were inconsistent with our research topic. After we read the full texts, twenty articles were ultimately included [6–25] (Table 1). Twenty-five articles were excluded because of duplicated cases; nine articles were excluded because newborns tested positive for COVID-19 had contacts with confirmed COVID-19 patients; two articles were excluded because the department of anesthesiology published a paper in which we suspected that these cases had appeared in other papers; and four articles were excluded because they were not related to parturition. The flow chart of publication selection is shown in Fig. 1. Eligible articles were mainly case reports and case series. The cases predominantly occurred around February 2020.

**Table 1:** Background characteristics of the selected studies

	Authors	Study date	Study location	Study design	Pregnancies
1	Zengyuan Yu et al. [6]	31 January, 2020	Zhengzhou, China	Case report	1
2	Ruihong Zhao et al. [7]	19 January, 2020	Hangzhou, China	Case report	1
3	Siyu Chen et al. [8]	20 January to 10 February, 2020	Wuhan, China	Retrospective cohort	5
4	Dong Hwan Lee et al. [9]	19 January, 2020	Daegu, Korea	Case report	1
5	Na Li et al. [10]	24 January to 29 February, 2020	Wuhan, China	Case–control study	16
6	Yang Li et al. [11]	12 January, 2020	Hangzhou, China	Case report	1
7	Xiaotong Wang et al. [12]	2 February, 2020	Suzhou, China	Case report	1
8	Zambrano LI et al. [13]	9 March, 2020	Tegucigalpa, Honduras	Case report	1
9	Lingkong Zeng et al. [14]	January to February, 2020	Wuhan, China	Retrospective cohort	33
10	Mengdie Li et al. [15]	29 January, 2020	Xinyang, China	Case report	1
11	Yiwei Zhao et al. [16]	31 January, 2020	Wenzhou, China	Case report	1
12	Renbin Zhou et al. [17]	12 February, 2020	Yichun, China	Case report	1
13	Siying Zhuang et al. [18]	20 January, 2020	Wuhan, China	Case report	1
14	E. Kalafat et al. [19]	20 March, 2020	Ankara, Turkey	Case report	1
15	Parisa Karami et al. [20]	31 March, 2020	Zanjan, Iran	Case report	1
16	Xiali Xiong et al. [21]	29 January, 2020	Beijing, China	Case report	1
17	Bin Zhang et al. [22]	1 February, 2020	Zhongshan, China	Case report	1
18	Liquan Huang et al. [23]	26 January, 2020	Wuhan, China	Case report	1
19	Lian Chen et al^.^ [24]	8 December, 2019, to 20 March, 2020	Wuhan, China	Retrospective cohort	118
20	Noelle Breslin et al. [25]	13 March to 17 March, 2020	New York, American	Retrospective cohort	43

Quality evaluation of all articles was performed according to several key questions [5]. For study selection, whether it was a case report or case series, each author collected the cases of which they were aware in their hospital, so the overall quality of this item was good. In the domain ‘ascertainment’, each case was diagnosed according to the latest clinical guidelines, so the overall quality of this item was the best. In terms of causality, these articles were mainly descriptive studies, so the overall quality was low. In the final domain of ‘reporting’, most articles provided as many details as possible, so the quality was good.

### Synthesis of the results

#### Maternal characteristics and outcomes

A total of 230 pregnant women with COVID-19 were included (Table 2). Five of them decided to terminate their pregnancy, four of which had induced abortions owing to the concerns about COVID-19. Three pregnant women had spontaneous abortions, and two patients had ectopic pregnancies. A total of 154 pregnant women delivered, and 66 pregnant women continued their pregnancy. Among these pregnant women with COVID-19, 25.00% of patients had chronic diseases, such as hypertension and polycystic ovary syndrome. A total of 34.62% of cases had obstetric complications, including anemia (31.58%), gestational hypertension (13.41%), preeclampsia (12.90%), and gestational diabetes (11.76%). The most common symptoms were fever (59.05%) and cough (54.76%), followed by postpartum fever (25.51%) and physical discomfort (21.43%). Myalgia, shortness of breath, headaches, and diarrhea were observed in 19 (12.75%), 20 (11.90%), 16 (11.35%), and 8 (5.06%) patients, respectively. For laboratory examinations, 57 (40.71%) patients developed lymphopenia, and 21 (16.84%) patients had leukocytosis. Platelet count was decreased in 5 (4.03%) pregnant women. Concentrations of transaminase, C-reactive protein, and D-dimer were elevated in 26 (25%), 83 (64.34%), and 92 (82.14%) patients, respectively. However, other parameters, though their significant prognostic values in COVID-19 were reported [26–28], were tested less frequently. Concentrations of procalcitonin (PCT), myocardial enzyme, and IL-6 were elevated in 5 (38.46%), 4 (44.44%), and 5 (100%) pregnant women, respectively. Reports of these three parameters included less than 10% of patients, and these characteristics should be interpreted with caution because of possible bias. Imaging revealed pulmonary infection in 97.12% of the patients.

**Table 2:** Maternal characteristics and outcomes

	Case numbers	Total numbers	%
Characteristics of patients			
Cesarean delivery	124	154	80.52
Vaginal delivery	30	154	19.48
Chronic diseases	21	84	25.00
Obstetric complications	18	52	34.62
Gestational hypertension	11	82	13.41
Gestational diabetes mellitus	4	34	11.76
Anemia	6	19	31.58
Preeclampsia	4	31	12.90
Clinical symptoms			
Fever	124	210	59.05
Cough	115	210	54.76
Myalgia	19	149	12.75
Sore throat	4	136	2.94
Physical discomfort	12	56	21.43
Shortness of breath	20	168	11.90
Diarrhea	8	158	5.06
Headache	16	141	11.35
Postpartum fever	25	98	25.51
Laboratory results			
Lymphocytes count < 1 × 10^9^ cells per L	57	140	40.71
The white blood cells count > 10 × 10^9^ cells per	21	124	16.84
Platelet count < 100 × 10^9^ per L	5	124	4.03
Transaminase concentration > 40 U/L	26	104	25.00
Myocardial enzyme > 190 IU/L	4	9	44.44
C-reactive protein concentration > 10 mg/L	83	129	64.34
IL-6 ≥ 7 pg/mL	5	5	100.00
D-dimer > 500 µg/L	92	112	82.14
Procalcitonin ≥ 0.25 ng/mL	5	13	38.46
Chest CT imaging			
Typical imaging manifestations	135	139	97.12
Treatment			
Oxygen support	67	112	59.82
Mechanical ventilation	7	135	5.19
Clinical classification			
Critical type	7	175	4.00
Severe type	14	175	8.00
Moderate type	144	175	82.29
Asymptomatic	10	175	5.71
Outcome			
Get well/discharge	229	230	99.57
Death	1	230	0.43

In terms of treatment, 7 (5.19%) pregnant women received mechanical ventilation, and 67 (59.82%) pregnant women received oxygen support through a nasal catheter. Most of the pregnant women received routine treatment, including antiviral drugs and antibiotics, to prevent infection. Only 7 (4.00%) pregnant women were critically ill, 14 (8.00%) women had severe cases, and 144 (82.29%) women had moderate disease. Ten (5.71%) pregnant women were asymptomatic. A critically ill pregnant woman with 35 weeks of pregnancy had septic shock, respiratory failure, and emergency cesarean section, and the newborn died of intrauterine asphyxia. This pregnant woman received invasive mechanical ventilation, extracorporeal membrane oxygenation (ECMO), continuous renal replacement therapy (CRRT), and convalescent plasma and ultimately survived [22, 29]. At present, the only deceased case is a 27-year-old woman with 30 weeks of gestation, who deteriorated rapidly and underwent invasive mechanical ventilation due to respiratory distress; the patient died of multiorgan failure. Her fetus was born with an Apgar score of 0 and without reaction to the neonatal cardiopulmonary resuscitation protocol [20]. Most of the pregnant women with COVID-19 were getting better or were discharged.

#### Perinatal characteristics and outcomes of newborns

A total of 156 newborns were born (Table 3). A total of 97.06% of newborns had a 1-min Apgar score above 8. All live newborns had 5-min Apgar scores above 8. A total of 24.74% of newborns were premature (24/97), and 5.45% of newborns had fetal distress (3/55). A total of 8.49% of newborns had premature rupture of membranes (9/106). Only one newborn received mechanical ventilation. Two newborns died unfortunately, including a newborn who died of intrauterine asphyxia [22, 29]; the other deceased newborn was born with an Apgar score of 0 and did not react to the neonatal cardiopulmonary resuscitation protocol [20].

**Table 3:** Perinatal characteristics and outcomes of newborns

	Case numbers	Total numbers	%
Apgar score (1 min) ≥ 8	99	102	97.06
Apgar score (5 min) ≥ 8	118	120	98.33
Premature	24	97	24.74
Fetal distress	3	55	5.45
Premature rupture of membranes	9	106	8.49
Mechanical ventilation	1	42	2.38
Oxygen therapy	0	10	0.00
Get well/discharge	154	156	98.72
Death	2	156	1.28

#### Possibility of vertical transmission

These 154 mothers with COVID-19 gave birth to 156 newborns (Table 4). The results of the nucleic acid test of SARS-CoV-2 in 128 newborns were reported, including 123 newborns’ negative throat swab tests and five positive tests [14, 15, 30]. Five infants who tested positive for SARS-CoV-2 were delivered by cesarean section. Elevated concentration of IgM and IgG against SARS-CoV-2 were detected in eight newborns whose throat swab test were negative [21, 31, 32], three of which had elevations in both IgM and IgG against SARS-CoV-2. Three newborns had elevated IgG but normal IgM. Interleukin 6 was also abnormally elevated in these six newborns. RT-PCR tests of SARS-CoV-2 of the vaginal secretions (*n* = 13), breast milk (*n* = 25), amniotic fluid (*n* = 32), placental blood (*n* = 35), and placental tissues (*n* = 9), specimens that are associated with vertical transmission, were all negative.

**Table 4:** Newborn SARS-CoV-2 nucleic acid detection and other specimens related

	Case numbers	Total numbers	%
Test for SARS-CoV-2 by nucleic acid	128	156	82.05
Negative result	123	128	96.09
Positive result	5	128	3.91
Via vaginal delivery	0	5	0
Via cesarean delivery	5	5	100
Test for SARS-CoV-2 by antibody	8	8	100
IgG increased and IgM increased	3	8	37.5
IgG increased and IgM normal	3	8	37.5
Vaginal secretions test negative	13	13	100.00
Breast milk test negative	25	25	100.00
Amniotic fluid test negative	32	32	100.00
Cord blood test negative	35	35	100.00
Placenta tissues test negative	9	9	100.00

## Discussion

### Main findings

The results of this systematic review showed that the clinical manifestations of pregnant women with COVID-19 were similar to those of general COVID-19 patients [33], including fever, cough, myalgia, shortness of breath, and diarrhea. The common laboratory changes of patients included lymphopenia, leukocytosis, decreased platelet counts, supraphysiological concentrations of transaminase, C-reactive protein, and D-dimer. The majority of chest CT scans showed typical imaging manifestations of COVID -19 pneumonia.

Common obstetric complications, such as gestational hypertension, preeclampsia, and gestational diabetes mellitus, may affect the outcomes. More than 80% of the patients were mild cases. Asymptomatic infections were reported in pregnant women. The proportion of mechanical ventilation was 5.19%, which was slightly lower than that of the general population [33]. The case-fatality rate (0.43%) is lower than the mortality of COVID-19 patients reported by World Health Organization (6.80%) and the Chinese Center for Disease Control and Prevention (2.29%) [34, 35] and similar to the overall maternal mortality rate worldwide (1 in 180) [36].

Most patients were in the third trimester of pregnancy. A total of 124 (80.52%) patients underwent a cesarean section. The most common adverse pregnancy outcome, premature delivery, occurred in up to 24.74% of pregnant women. Premature rupture of membranes, fetal distress, and even fetal death were also reported.

There was more evidence for vertical transmission of COVID-19. Under strict protection during delivery and postpartum isolation measures, 3.91% (5/128) of newborns were tested positive for SARS-CoV-2 nucleic acid, and elevation of IgM against SARS-CoV-2 was found in 3/8 newborns with negative of SARS-CoV-2 nucleic acid results. However, more details, including nucleic acid test results of vaginal secretions, breast milk, amniotic fluid, placenta tissues, and cord blood, did not support vertical transmission. In particular, newborns with a positive SARS-CoV-2 nucleic acid test or elevated serum antibody were all delivered by cesarean section. From the perspectives of disinfection and protection, breastfeeding may not cause neonatal infection.

### Strengths and limitations

Several reviews have summarized the situation of pregnant women infected with COVID-19 [37–39], however, knowledge needs to be summarized in a timely manner. The strengths are as follows. First, the databases were comprehensive; therefore, we enrolled as many timely articles as possible. Second, we carefully and strictly reviewed articles to remove as much duplicated data as possible. Finally, we summarized comprehensive details related to vertical transmission.

One of our limitations is the inclusion of case reports, which may be inclined to report more severe cases. Another limitation is that all included articles were retrospective studies. Prospective studies will lead to a better understanding of pregnant women with COVID-19.

### Interpretation

Pregnant women are at high risk of infection or may be already infected with COVID-19. Psychological stress and viral infections are both risk factors for miscarriage [40]. The psychological pressure brought by COVID-19 has driven several patients to terminate their pregnancies [24]. More information about pregnant women with COVID-19 is urgently needed to relieve their anxiety and guide them to make beneficial decisions.

According to our results, most patients are asymptomatic or mildly ill. They come to the hospital because of their pregnancy and the need for medical care for childbirth. Then, the detection of coronavirus infection reveals that these patients are in an early stage of infection, with milder symptoms, or may even be asymptomatic or have very few symptoms, which may be one of the contributors to their favorable prognosis. However, asymptomatic infections do not seem to be a minority. A report of new cases indicates that up to four-fifths of people are asymptomatic [41], and 32.6% of pregnant women with COVID-19 had no symptoms in a New York hospital [25]. Isolation at home and travel restrictions protect people from infection but disrupt routine prenatal examinations and the discovery of abnormal pregnancies in time. Pregnant women are in states of anxiety and worry regarding issues such as the epidemic’s status, the impacts on maternal and child health, the safety of CT examination, and treatment prognosis for the fetus and the mother [42]. Pregnant women may be worried about being infected and may demand psychological consultation [43]. We agree that measures such as pregnant women being screened and followed up, being offered more flexible birth inspection strategies [44], and receiving more concern for their mental health are required [45].

SARS infection during pregnancy has been correlated with a high incidence of spontaneous abortion and premature delivery [37, 46]. Similar phenomena have also been observed in pregnant women with COVID-19. In three placental pathology reports, no pathological alternation of villitis and chorioamnionitis was observed [47]. To understand the underlying mechanism, more placental pathological reports, especially those with adverse neonatal outcomes, are needed.

Regarding delivery mode, cesarean section and vaginal delivery have their respective short- and long-term advantages and disadvantages [48, 49]. During vaginal delivery, amniotic fluid, vaginal bleeding, and vaginal discharge increase the difficulty of infection control. Cesarean section seems to benefit both patients and medical staff [50], but there is no clear evidence on whether vaginal delivery or cesarean section is more beneficial [51]. This review shows that more pregnant women with COVID-19 delivered by cesarean section instead of via the vagina. On the one hand, a high incidence of intraoperative hypotension has been noted in pregnant COVID-19 patients during cesarean section [52, 53]; on the other hand, no evidence of vertical transmission was observed with vaginal delivery.

About a decade ago, the limited publications on pregnant women with SARS reported no evidence of vertical transmission [3, 54]. Here, we included some single-center studies to summarize the possibility of vertical transmission of COVID-19 [30, 31, 47, 55–58]. Although strict isolation measures are taken, a few newborns still show positive results on the SARS-CoV-2 nucleic acid test. Possible mechanisms for vertical transmission (from the mother to the fetus or newborn) mainly include: trans-placental transmission after the virus infects the placenta, intrapartum transmission via ingestion or aspiration of cervical vaginal secretions during delivery, and postpartum transmission by breastfeeding [59]. Evidence supporting intrauterine transmission is the discovery of elevated IgM and IgG against SARS-CoV-2 in infants [31, 32]. Unlike IgG, IgM is not usually transferred from mother to fetus because of its larger macromolecular structure. Therefore, it is probably produced by the fetus after infection. Vertical transmission could be considered when both IgM and IgG are elevated conservatively. Paradoxically, angiotensin-converting enzyme 2(ACE 2), the receptor of SARS-CoV-2, is poorly expressed in various cell types at the maternal–fetal interface [60], and SARS-CoV-2 was undetected in the placenta histopathology of infected parturients [47]. Though the evidence is inconclusive, no vertical transmission phenomenon or evidence of the vaginal delivery process has been found. According to the cautious advice of experts [61, 62], the vast majority of newborns were separated from their COVID-19 confirmed mothers and fed formula milk powder. The WHO recommends that mothers can share a room with their infants and provide breast feeding after SARS-CoV-2 testing is negative in their breast milk [63]. Mothers should wear masks, practice hand hygiene [64], and disinfect all containers of expressed human milk [65].

Only 6.5% of the newborns with negative PCR results were also tested for serum antibodies against SARS-CoV-2. It will not be representative and convincing to calculate the vertical transmission probability with such a small sample size and possible biased data. One study showed that the sensitivity of RT-PCR to detect viral nucleic acids was only 71% [66]. Collecting a variety of samples, in addition to nasopharyngeal swabs, can improve the positive detection rate [67]. Serum antibodies against SARS-CoV-2 should be tested more frequently and multiple samples should be included in pathogenic testing.

## Conclusion

This study included 230 pregnant women, of which 154 delivered 156 newborns, and summarized the clinical characteristics and maternal and neonatal outcomes of pregnant women with COVID-19. Most patients had a mild illness. Symptoms, chest CT imaging, and laboratory tests of infected pregnant women were similar to those of the overall patients with COVID-19. Cesarean section was more common than vaginal delivery in COVID-19 parturients. Worries about infection led to several abortions. Premature delivery was the most common adverse pregnancy outcome. The maternal mortality rate was 0.43%, and the neonatal mortality rate was 1.28%. The vertical transmission rate calculated by the PCR-proven COVID-19 test was 3.91%, and all five newborns with positive results were delivered by cesarean dissection. Other related findings we summarized do not provide more evidence for the vertical transmission of SARS-CoV-2. Serum antibodies against SARS-CoV-2 should be tested more frequently, and multiple samples should be included in pathogenic testing.

## Data Availability

Data and material were available.
